# Effect of Changing Climatic Conditions on Properties of Wood Textile Composites

**DOI:** 10.3390/ma18122764

**Published:** 2025-06-12

**Authors:** Claudia L. von Boyneburgk, Hans-Peter Heim

**Affiliations:** Institute of Materials Engineering, Plastics Engineering, University of Kassel, 34125 Kassel, Germany

**Keywords:** wood–textile composites, materials testing, artificial weathering, FTIR

## Abstract

Wood–textile composites (WTCs), consisting of polypropylene and woven willow wood, have potential for both interior and exterior applications. However, their basic materials are not inherently resistant to outdoor weathering. This study examines the impact of various climatic conditions on the material behavior of WTCs. The composite and its components were aged under different scenarios, including kiln-drying, frost, standard and tropical climate, and artificial weathering and water storage, and analyzed for dimensional stability, chemical changes (FTIR), mechanical damage (µ-CT), and mechanical performance. While kiln-drying, frost, and tropical climates had only minor effects, water storage caused swelling-related damage, resulting in a 45% decrease in Young’s modulus but increased elongation at break (+88%) and impact strength (+75%). Artificial weathering led to significant degradation: tensile strength declined by 28%, Young’s modulus by 49%, and impact strength by 26%. In the medium term, this degradation compromises the integrity of the composite. The results highlight the need for effective stabilization measures—such as polymer modification or structural protection—to ensure the long-term durability of WTCs in outdoor use.

## 1. Introduction

Wood–textile composites (WTCs) are a new class of materials in the field of natural fiber-reinforced composites, inspired by the idea of textile architecture [1,2,3] and the desire to increase the proportion of renewable raw materials in the field of materials, especially against the backdrop of a circular economy [4,5]. WTCs are defined by the embedding of solid wood fabrics in a thermoplastic matrix. The fabrics consist of solid wood strips (quasi-endless with variable cross-sections), which in this study were obtained from branches of the willow species *Salix americana*. The wood strips are processed into fabrics on a specially adapted loom, with a wide range of options available in terms of the spacing between the strips, the weave, and the resulting weave patterns [5].

Depending on their area of application, composite materials face a lot of challenges. While these are initially negligible at the mechanical level when used for neat interior design elements, a wide range of mechanical and thermo-hygroscopic influences arise when they are used outdoors. Unlike the glass fiber fabrics (cross-section of individual fibers: 3.5–24 μm; cross-section of rovings: 0.3–0.8 mm) or carbon fiber fabrics (cross-section of individual fibers: 5–10 μm; cross-section of rovings: 0.5–2.5 mm) regularly used to reinforce thermoplastics [6,7], the strips of the wood fabrics, with a cross-section of approx. 1 mm × 4 mm, are significantly oversized in comparison. Although the wood strips used are composed of many individual fibers and filaments, similarly to textile rovings, wood is a fiber composite material by itself, consisting of the structural substances cellulose, hemicellulose, and lignin [8]. Due to this, the anisotropic properties of the wood fibers used and their influence on the composite material must be taken into account.

The use of solid wood in woven form has only been investigated by Haghdan et al. [9] to date, who investigated the impact behavior of wood–polyester composites reinforced with woven Douglas fir veneer. Similarly to natural fiber fabrics, the woven structure significantly improved impact resistance compared to non-woven veneers. The WTCs analyzed in this study have previously mainly been studied under standard climatic conditions, focusing on their mechanical performance and comparability with conventional fiber-reinforced plastics. Their mechanical properties were found to be comparable to those of jute fabric-reinforced thermoplastics, and they fulfill the typical requirements of fiber composites. However, the results also highlighted that the natural fiber architecture of the wood plays a critical role in its failure behavior and must be considered in the simulation and design of components [4,10,11,12]. The characterization of WTCs when used in unprotected outdoor areas has not yet been the subject of scientific investigation.

The present study aims to investigate the influence of various weather conditions on WTCs in their original form, i.e., without additives. Composites based on wood and thermoplastic polymers are significantly more susceptible to photo-oxidative degradation than the neat basic materials, especially when wood is used in the form of particles or fibers. This susceptibility increases with the wood content in the composite and is particularly pronounced when exposed to water [13,14]. When using polyolefins as a matrix, it must be considered that these are also photochemically unstable and must therefore be modified with UV stabilizers for outdoor use. There are various options for this, such as pigments, UV absorbers, or HALS (hindered amine light stabilizers) [15,16]. In wood–plastic composites (WPCs), the wood content causes the particles near the surface to turn gray due to the free radicals that are produced during the photo-oxidative degradation of the lignin [17]. If water is added to the photo-oxidative degradation of the wood, the wood particles will swell and shrink. This leads to microcracks in the interface between the wood and the polymer, which usually results in a deterioration in the mechanical properties [18,19]. The resulting cracks and degradation of the polymer also increase the surface roughness, thereby enlarging the area exposed to attack. The exposed wood particles on the surface cause a kind of wick effect, which allows additional water to penetrate the composite material. With prolonged exposure, this also increases susceptibility to fungi and, as a result, biodegradability [20,21,22]. In addition to modifying the polymer matrix, modifying wood can also influence the stability of the composite material under varying weather conditions. As with polymers, there are various approaches to increase stability to weather, such as removing extractives or lignin, or bonding a UV absorber to the wood surface. In addition, the choice of wood species also influences the velocity of degradation [23].

While quasi-static tests have shown that an increase in moisture in the composite leads to a decrease in mechanical properties, its effect on these properties under dynamic impact stress is unclear. Friedrich showed [24] that the properties of WPC facade elements significantly deteriorated under bending tests after one year of outdoor use in a Central European climate. Bledzki et al. [25], on the other hand, showed that increasing moisture alone did not result in a significant decrease in impact strength. In the impact test, there was even an increase in the maximum force with increasing moisture content. This was further enhanced by the use of longer wood fibers and the associated increase in water absorption. The effects of aging on the properties of composite materials are therefore extremely complex, with the choice of materials and the duration of exposure playing a particularly important role.

**Figure 1:**
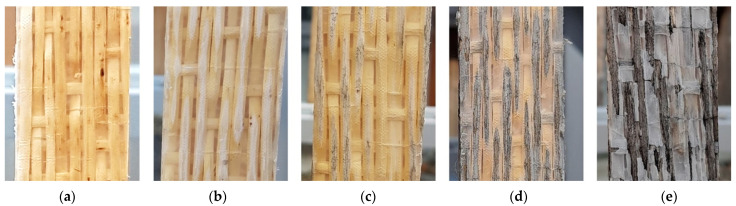
Progression of weather-induced degradation of a WTC when exposed to outdoor weathering: (**a**) after 0 months; (**b**) after 2 months; (**c**) after 4 months; (**d**) after 10 months; (**e**) after 17 months.

If the WTCs examined here are used in unprotected outdoor areas, serious weather-related degradation of the composite material occurs, as shown in Figure 1 for 2, 4, 10, and 17 months of outdoor expose. After just 17 months of outdoor weathering (Central Europe, Germany, Kassel: moderate mid-mountain climate), the plastic matrix is almost completely destroyed, while the wood shows severe photochemical and biotic degradation.

The aim of this study is to investigate the effects of various climatic conditions on the properties of WTCs in detail. To this end, the influence of drying, frost, standard climate, tropical climate, artificial weathering, and water storage is analyzed to create a comprehensive understanding of potential degradation scenarios. The focus is on assessing thermo-hygroscopic effects on both the chemical and mechanical behaviors of the base materials and the resulting composite. The overarching goal is to identify fundamental degradation mechanisms and derive recommendations for stabilizing WTCs for use in unprotected outdoor environments.

## 2. Materials and Methods

### 2.1. Basic Materials and Manufacturing

For the production of the fabrics, willow branches (*Salix americana*) were processed into willow wood strips with a cross-section of approx. 4 mm × 1 mm. Since testing perpendicular to the fiber direction is not possible on these strips and testing in the fiber direction is only possible to a limited extent, over the course of this study, solid willow wood was additionally used, which was processed into test specimens using a laboratory circular saw. The matrix mainly consists of neat PP (Sabic^®^ PP 520P, SABIC SALES Europe B. V., Sittard, The Netherlands), with 5 wt.% maleic anhydride-grafted PP (MAH-PP) (Licocene PP MA 6452, Clariant International Ltd., Muttenz, Switzerland) incorporated to improve adhesion in the areas in contact with the wood.

For the production of WTCs, the willow wood strips were processed into fabrics on a specially modified loom [5]. In this case, the atlas weave (1:7) was used, which produces different weaving patterns on the top and bottom sides and provides the fabric with anisotropic properties. The atlas weave was chosen because it has significantly fewer crossing points than, for example, a plain weave, while using the same amount of material. Although this results in a slightly lower displacement resistance, the reduced number of crossing points ensures excellent drapability, as the overall stiffness of the fabric is reduced. At the same time, the mechanical strength is increased [7]. In a subsequent hot compacting process, two fabrics are draped with their bottom sides facing each other, but with the same main fiber direction, and coated with a thermoplastic matrix. The matrix consists of twelve parts neat PP and four parts PP, with 5 wt.% MAH-PP on the wood surfaces. The finished composite material consists of 60 wt.-% matrix and 40 wt.-% wood. Sheets with a size of 500 mm × 500 mm and a thickness of 4 mm were produced at a maximum temperature of 180 °C, a maximum pressure of 0.32 MPa, and a maximum residence time of 30 min including cooling time. A detailed description of the manufacturing process of WTCs can be found in [4]. The WTC specimens, as well as the willow specimens made from solid wood required for the mechanical characterization, were cut out of plates using a laboratory circular saw. The neat PP test specimens were injection molded in accordance with the relevant standards. All specimens where stored at 23 °C and 50% r. h. in a standard atmosphere according to EN ISO 291 [26] until they were tested.

All results obtained in the course of this study were examined for significance using a *t*-test with the assumption of unequal variances. In addition, the results were checked for stability using the coefficient of variation (CV).

### 2.2. Aging of Specimens

Table 1 shows the aging conditions selected for the weathering of WTCs and their basic materials. The aim was to simulate, as comprehensively as possible, the weather conditions to which WTCs could be exposed during outdoor applications in a Central European climate. For aging in a standard climate and in a tropical climate, the test specimens were stored in a WK1-340/70 climate chamber from Weiss Technik GmbH (Reiskirchen, Germany) under the specified conditions for 168 h [26]. The kiln-drying was carried out in a convection oven. The storage in the frost was carried out in a freezer, whereby the temperature in the area of the stored samples was checked with an additional thermometer. Water storage was carried out in a water bath with an immersion device; excess water was dabbed off after removal and before testing the samples [27]. The artificial weathering was realized in a climate chamber UV 200 SB from Weiss Technik GmbH (Reiskirchen, Germany), in accordance with EN ISO 16474-3 [28]. The total cycle length was 6 h, during which the samples were exposed to UV radiation at 50 °C for five hours, and immediately after this they were exposed to a one-hour sprinkling at 23 °C. This process was repeated eighty-four times, resulting in a total weathering duration of 504 h. With the exception of the weathered samples, which were stored in a standard climate for at least 24 h after weathering, all samples were tested immediately after removal from the respective climate.

### 2.3. Thermal Expansion

One aspect of thermo-hygroscopic influences is the change in the dimensions of materials with changing ambient temperature. In view of the very different thermal expansion coefficients α_th_ of wood (2.5–5.0 × 10^−6^ K^−1^ [30]) and polypropylene (200 × 10^−6^ K^−1^ [31]), the materials used were examined independently of each other with regard to their behavior at changing ambient temperatures. A device for dynamic mechanical analysis (DMA module Q 800, TA Instruments, New Castle, DE, USA) was used, which is able to measure the change in the length of the clamped test specimen as a function of the surrounding temperature. For this purpose, test specimens were mounted in the tensile test clamp of the device used. PP samples were prepared from injection-molded tensile test specimens (20 mm × 10 mm × 4 mm), and sections of the willow strips in the fiber direction (10 mm × 4 mm × 1 mm) were separated from the willow strips. Subsequently, a stabilization temperature of 23 °C was initially applied and held isothermally for 15 min. Based on the parameters given in Table 1, the temperatures of the respective aging conditions were then approached with a temperature ramp of 3 K/min. Each temperature was held isothermally for 15 min after reaching the target temperature to allow the dimensions of the test specimens to adjust to the respective temperature. The current linear expansion of the test specimen was determined at each temperature.

### 2.4. Swelling and Shrinking

Thermal expansion usually occurs in conjunction with ambient humidity. Hygroscopic materials such as wood are particularly affected, but polymers can also swell and shrink. These effects, like changing temperatures, lead to dimensional changes in the basic materials, which also affect the composite. For a more precise investigation, injection-molded test specimens made of PP and sawn test specimens made of willow wood and a WTC measuring 80 mm × 10 mm × 4 mm were prepared and initially stored in a standard climate for 7 days. After this, the samples were weighed and aged according to the parameters given in Table 1. Immediately after aging, the weight was determined again. The change in the moisture content (MC) of the aged samples, based on the MC in the standard climate [27,29], was determined using Formula (1), with c as the relative change in the mass of the test specimen; m_1_ representing the mass of the test specimen after aging in standard climate (mg); and m_2_ representing the mass of the test specimen after aging (mg).

(1) $$c=\frac{m_{2}-m_{1}}{m_{1}}\times 100\%$$

In addition to determining their weight, the test specimens of the basic materials were measured both before and after aging in terms of their thickness and width, and in the case of willow wood, also in terms of length. The measurement was performed using a caliper with a precision of two decimal places. The relative change in dimension per direction was also determined according to Formula (1), which was adapted as follows: m_1_ = dimensions of the test specimen after storage in standard climate (length, width, or height, in mm); m_2_ = dimensions of the test piece after aging (length, width, or height, in mm).

### 2.5. μ-CT Measurements

In order to visualize weather-related changes inside the WTCs, µ-CT images were taken using a computer tomograph (Zeiss Xradia Versa 520 µ-CT, Carl Zeiss AG, Oberkochen, Germany). Aged WTC test specimens were examined for any damage that may have occurred during the aging process.

### 2.6. Color Measurements

For an accurate detection of possible color changes, a color spectroscopy was performed on the aged WTC samples using the UltraScan-Pro spectrophotometer from Hunterlab (Murnau, Germany), which determines the color of a material in the L*a*b* color space (L = light–dark; a = red–green; b = blue–yellow). First, the changes in absolute values were compared to the standard climate. Next, the color difference, Δ*E*, was determined using Formula (2), where Δ*L*, Δ*a,* and Δ*b* represent the changes in the L*a*b* values after aging relative to the standard climate:

(2) $$\Delta E=\sqrt{\Delta L^{2}+\Delta a^{2}+\Delta b²}$$

### 2.7. FTIR

For the detection of chemical changes in the material surface, FTIR measurements were performed on the aged composite samples using a Fourier Transform Infrared Spectrophotometer IRAffinity-1S from Shimadzu (Kyoto, Japan). The recordings were made in the wavelength range between 500 nm and 4000 nm with a resolution of 4 cm^−1^ and an average of 45 scans. The measurements were evaluated using LabSolutions IR Version 2.24 software (Shimadzu Corporation, Kyoto, Japan). The spectra determined for PP were normalized at 974 cm^−1^ [32], while those for willow wood were normalized at 2050 cm^−1^ [33] and smoothed, followed by a multipoint baseline correction.

Indices can be calculated to provide a detailed evaluation of the FTIR measurements. This was achieved using Formula (3), with the formula for the carbonyl index of PP shown here as an example [32]:

(3) $$\Delta Carbonyl-Index=\left(\frac{A_{1715\;cm-1}}{A_{974\;cm-1}}\right)aged-\left(\frac{A_{1715\;cm-1}}{A_{974\;cm-1}}\right)not\;aged$$

However, further indices were determined for both willow wood and PP; the respective wavelengths used for the calculation are listed in Table 2.

### 2.8. Mechanical Testing

To assess the influence of aging on the mechanical properties, tensile tests and impact bending tests were carried out on the aged test specimens.

The tensile test was carried out in accordance with EN ISO 527-4 [48] on a universal testing machine from ZwickRoell GmbH & Co. KG (Ulm, Germany). For this purpose, WTC test specimens were produced in the main fiber direction (type 2 according to DIN EN ISO 527-4: 250 mm × 25 mm) and aged according to the aging conditions specified in Table 1. The test speed was 5 mm/min, 10 test specimens were tested from each material, and the tensile strength, modulus of elasticity, and elongation at break were evaluated.

The instrumented impact bending test was performed in accordance with EN ISO 179-2 [49]. For this purpose, an instrumented Charpy impact pendulum (ZwickRoell GmbH & Co. KG, Ulm, Germany) with a 5 J hammer was used. The dimensions of the test specimens corresponded to type 1fUc according to EN ISO 179-1 [50]. Ten test specimens were produced from each material in the main fiber direction, aged according to the aging conditions specified in Table 1, tested, and evaluated to determine their impact strength.

## 3. Results and Discussion

### 3.1. Thermal Expansion of the Basic Materials

**Figure 2:**
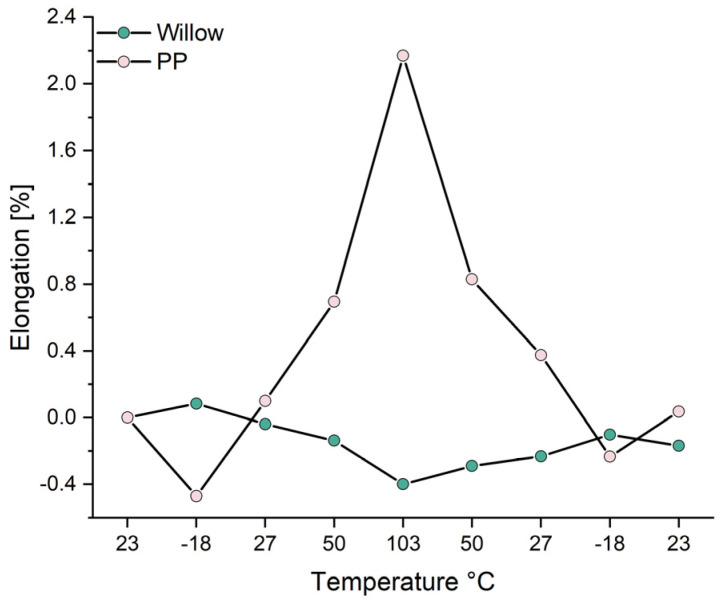
Relative thermal expansion of willow strips and PP specimens.

Figure 2 shows the relative change in the length of willow wood and PP as a function of the surrounding temperature, detected using DMA measurements. The results confirm the assumption that the materials used exhibit very different behaviors when the surrounding temperature changes. On the one hand, the length of the polymer changes more than that of wood, as expected; on the other hand, the change in length of the polymer follows the temperature, while the change in length of wood occurs in the opposite direction. In addition to temperature, the absorption or release of moisture from or into the surrounding air must always be taken into account, which also has different effects on the two materials. While the surrounding humidity has only a slight influence on the non-polar PP, the absorption and release of humidity plays a decisive role in the case of hydrophilic wood. Thus, an increase in temperature leads to the release of water, which explains the shrinkage of the test specimens at 103 °C [51]. When freezing, on the other hand, the length of the willow strip increases, which can be explained by the freezing and the associated expansion of the water contained in the wood [52]. For the composite material, the behavior of the two materials means that a change in the ambient temperature can lead to stress between the joined materials, especially at the interface. In theory, such effects may cause cracking and delamination, weakening the composite even in the absence of external mechanical stress. However, the polypropylene used has an elongation at break of approximately 6.5% [12], whereas the thermally induced length changes determined by DMA were just over 2% of the total. This suggests that ambient temperature fluctuations alone are unlikely to cause initial damage to the composite.

### 3.2. Thermo-Hygroscopic Effects on Basic Material and Composite

In order to investigate the additional influence of moisture on the dimensions mentioned in Section 3.1 in more detail, the moisture content (MC) of the basic materials and, simultaneously, the changes in dimensions were determined.

**Figure 3:**
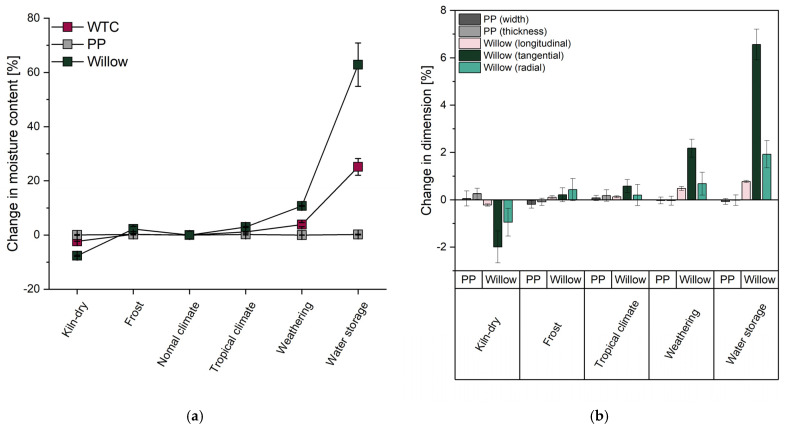
Thermo-hygroscopic effects: (**a**) relative change in the MC of PP, neat willow wood, and WTC and (**b**) relative dimensional changes in the test specimens of PP and neat willow wood, in each case as a function of the ambient climate.

The determined increase or decrease in the MC of the materials depending on the respective aging is shown in Figure 3a, with the MC in the standard climate serving as a reference in each case. In addition to the basic materials, PP, and willow wood, the WTC was also examined. When considering the results of the PP, it was initially assumed that no changes had occurred due to the low polarity. However, the significance analysis showed that storage in frost, in a tropical climate, and in water led to a highly significant increase in material MC of about 0.2%. Similar values were determined, for example, by El Hachem et al. [53] for isotactic polypropylene and by Robertson et al. [54] for LDPE. Since polypropylene, like all olefins, is considered hydrophobic, the very low water absorption, despite its significance, is due to diffusion and permeation processes in the amorphous areas and in defects in the crystalline areas [55]. In the case of the hydrophilic willow wood, however, all forms of aging caused a highly significant change in MC. Kiln-drying, for example, caused a decrease in moisture of almost 8%, while storage in frost and in a tropical climate caused an increase of between 2% and 3%. The increase in MC after water storage, was over 60%, which means that a large proportion of the cavities in the wood are also filled with water beyond the fiber saturation point.

Similarly to the basic materials, the MC of the WTC test specimens decreased significantly during kiln-drying, although the decrease was significantly lower compared to that of neat willow wood. In contrast to the basic materials, no significance could be detected for the increase in moisture during storage in frosty conditions; the same was true for the absorption of moisture in the tropical climate. The test specimens stored in water showed a moisture content of almost 25% after 24 h of water storage, which is close to the fiber saturation range of the wood [30]. This represents a significantly lower water absorption than that determined for the neat willow wood in the same amount of time. It can be assumed that the composite with a polymer matrix partially hinders the water absorption of the wood. Thus, water absorption can initially only take place in the peripheral areas that are not surrounded by the polymer, with the highest absorption occurring via the end faces of the wood. Here, the water can penetrate in both the radial and tangential directions, and the exposed lumens of the wood absorb water [54]. This leads to swelling and shrinking effects at the cut edges perpendicular to the main fiber direction, while the inner areas of the test specimen are initially protected from the influence of aging by the composite with the polymers. This results in a reduction in moisture absorption and release compared to unprotected wood [8,56]. Compared to the neat PP, however, the composite with wood undergoes a significant increase in water absorption and water release.

When evaluating the weathered specimens in the climate chamber, it must be considered that the moisture content depends on the removal time, as humidity cycles within the chamber. Immediately after the one-hour rain phase, the moisture content of both the willow wood and WTC specimens was near the fiber saturation point. However, since specimens were removed during the subsequent drying or UV phase and then stored for 24 h under standard climate conditions, their actual moisture content at the time of testing was lower. Although the trends shown in Figure 3a reflect expected behavior, no direct comparison can be made with other aging scenarios. This is further complicated by additional factors such as the chemical degradation of the wood and polymers, which will be addressed in later sections.

The dimensional changes in PP and willow wood depending on the level of aging are shown in Figure 3b, based on the reference at standard climate. As expected from the evaluation of the relative material moisture, the relative dimensions of the examined PP test specimens also show significant changes depending on the selected level of aging. Only the weathered test specimens showed no significant changes, which can be attributed to the sampling time in the climate chamber, as mentioned above, and to further degradation effects, which will be described more detailed below. The determined dimensional changes in the PP also led to significant differences in width and thickness. However, the thermal anisotropy of injection-molded PP parts, which depends, among other things, on the flow direction and the resulting crystallinity, is known from the literature [57,58]. Based on the planned processing using the hot-compacting process, and with the addition of willow wood fabrics, it can be assumed that the anisotropy determined for the injection molded test specimens will have only a minor influence on the hot-compacted composite. The kiln-dried PP samples showed the greatest dimensional change compared to the other samples. However, due to the significant but basically very small change observed in the dimensions of PP due to water absorption, it can be assumed that the change in ambient temperature has a greater impact than a change in ambient humidity for these polymers.

In the case of the willow wood examined, significant dimensional changes were observed in all directions measured, as was to be expected due to its hygroscopic nature. With the exception of storage in frosty conditions, the smallest change occurred in the fiber direction, i.e., in the longitudinal direction, for all the aging conditions examined. In the thickness direction and radial direction, dimensional changes occurred at medium levels, while the most significant swelling and shrinkage occurred in the cross-fiber direction and tangentially. This behavior, as well as the determined values, corresponds to the anisotropic behavior of wood when absorbing or releasing water, as is known from the literature [51,59].

The results of the measured thermal expansion and dimensional changes due to thermo-hygroscopic effects highlight a fundamental issue that particularly affects composite materials in which at least one component is sensitive to changes in ambient humidity and temperature. In the WTC examined here, both base materials respond to variations in both temperature and humidity. While the impact of temperature is more pronounced in PP, humidity has a greater effect on willow wood. The most immediate consequences are often visual, such as the appearance of visible cracks. However, for materials that swell or shrink significantly—like wood—these dimensional changes can also lead to more serious problems, such as the failure of adhesive bonds or the loosening of press-fits, potentially damaging or rendering components unusable. When combining materials with very different sensitivities, as in this case, the strongly divergent responses of the individual materials become a critical factor [51,60]. With the exception of the water-saturated specimens, none of the measured dimensional changes—like those previously identified through DMA measurements—approach the strain at break of PP, which is 6.5%. It can therefore be assumed that, under the aging conditions investigated, neither temperature changes alone nor in combination with variations in ambient humidity will cause damage to the composite.

The situation is different, however, with the specimens stored in water. Compared to injection-molded natural fiber-reinforced thermoplastics, the WTCs examined here show significantly increased water absorption and a dimensional change that exceeds the elongation at break of the PP used as the matrix material. Robertson et al. [54] examined the water absorption of natural fiber-reinforced LDPE with different natural fiber types, determining a maximum water absorption of 13.5% over several weeks. However, they were able to demonstrate that water absorption increases with the fiber content and also depends on the size of the fibers themselves. Very small and uniform fibers showed significantly lower water absorption than larger, coarser fibers, even when an identical fiber content was used. The authors attributed these differences to a better fiber–matrix connection and a more homogeneous distribution of the finer particles in the matrix, which significantly reduces the transport paths for water absorption from fiber to fiber. In addition, they assumed that for larger natural fibers, the internal transport paths (lumens) contributed to an increase in water absorption. Based on the significantly increased dimensions of the willow wood strips used here, there is a significantly increased number of lumens in the WTC, which leads to a comparative increase in the water absorption of the composite.

**Figure 4:**
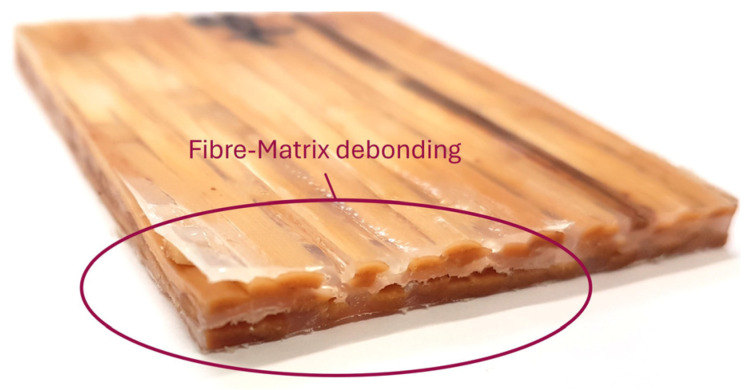
WTC test specimens after storage in water for 24 h.

Figure 4 shows a WTC test specimen after 24 h of water storage. As expected, the willow wood fibers in areas not surrounded by a plastic matrix have swollen considerably. The resulting swelling of the wood led to shear stresses in these areas, causing the plastic matrix to delaminate from the wood. This, in turn, creates further areas where the water comes into direct contact with the wood and thus leads to further penetration. Although the plastic matrix can delay the penetration of water, which can be seen in this case from the still light-colored central areas of the test specimen that were not penetrated by water, it is not possible to permanently prevent the composite from being saturated with water.

### 3.3. Results of the μ-CT Measurements

After aging the test specimens, external damage was only visible on the weathered and water-stored specimens. CT measurements were carried out to check whether this could also be confirmed inside the specimens. These initially showed that the specimens dried in the oven and stored in frosty and tropical climatic conditions did not exhibit any visually detectable changes. The water-stored sample also showed no changes in the area in the middle of the specimen from which the sample was taken, as the water had not penetrated this part of the sample after 24 h. In contrast, the weathered sample also showed visually detectable damage on the inside.

**Figure 5:**
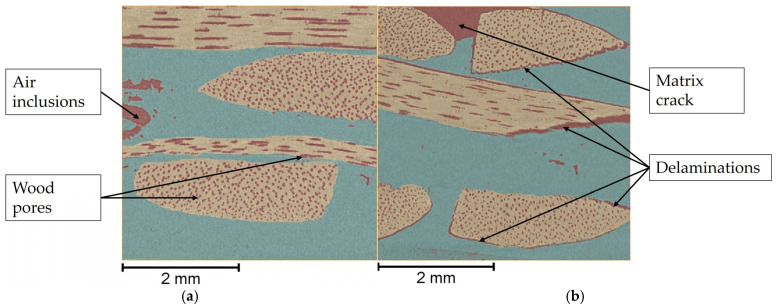
CT images of WTC after storage (**a**) in a standard climate and (**b**) after weathering.

This is shown in Figure 5, which presents a test specimen after storage in a standard climate (a) and a test specimen after weathering (b). The turquoise areas represent the matrix while the wood strips are shown in beige. All air-filled areas, both within the matrix and within the wood, are shown in red. In the case of the test specimen stored in a standard climate, a form-fitting distribution of the matrix around the willow strips can be seen, which indicates a good fiber–matrix connection without any defects. In the area of the matrix, some air-filled spaces can be seen, which can be traced back to air that entered the matrix during the hot-compacting process. In contrast, air-filled spaces can be seen around almost all the wood strips in the weathered test specimen, as well as matrix cracks. These effects result from the detachment of the matrix from the fibers in the course of weathering, triggered by the unequal stress states of the two basic materials described in Section 3.1 and Section 3.2. Similar findings were obtained by Sit et al. [61] in their investigations of weathered, flax fiber-reinforced PLA, where fiber fractures were visible in addition to delamination and matrix cracks. Fiber fractures could not be determined in the case of the WTC examined here due to the significantly larger dimensions of the willow strips and their resulting higher stability under environmental conditions. The µ-CT images therefore confirm the assumption that the composite material was damaged by weathering to such an extent, even inside, that a reduction in mechanical properties can be expected without previous mechanical stress.

### 3.4. Results of the Color Measurements

No color changes were visible to the naked eye in frozen or kiln-dried WTC-samples or in samples stored in a tropical climate compared to the standard climate. In contrast, a clear change in appearance was evident in the water-stored and weathered test specimens during the visual inspection of the test specimens. Table 3 shows the absolute L*a*b* values of the color measurements, including the corresponding CV. With the exception of the a* and b* values of the weathered samples, all values determined show little variation and can therefore be assumed to be stable.

The L* value showed a significant decrease in the case of the water-stored sample, which can be attributed to the darkening of the wood as a result of water absorption. In this process, liquid water penetrates the lumens of the wood, causing less light to be reflected [62]. The L* value showed a marginally significant decrease in the case of the air-dried sample, which can be attributed to the darkening of the wood during drying [63]. In the case of the weathered sample, the L* value showed a slight but not significant increase. Similar studies have shown that this increase is due to photochemical changes in the surrounding PP and the loss of its transparency, as wood normally darkens during weathering [64]. The b* value changes significantly in the case of the weathered sample. An increase in the b* value could result from yellowing caused by the formation of secondary chromophore groups and represents a visible, short-term effect of the onset of lignin degradation. As the process continues, UV degradation causes double refraction in the amorphous cellulose while selectively preserving the crystalline cellulose. Compared to the standard climate, the b* value decreases significantly, as is the case with the test specimens examined here. In the final phase of degradation, the chromophore structures are washed out of the wood, resulting in the silver or gray surface patina on the wood [65].

**Figure 6:**
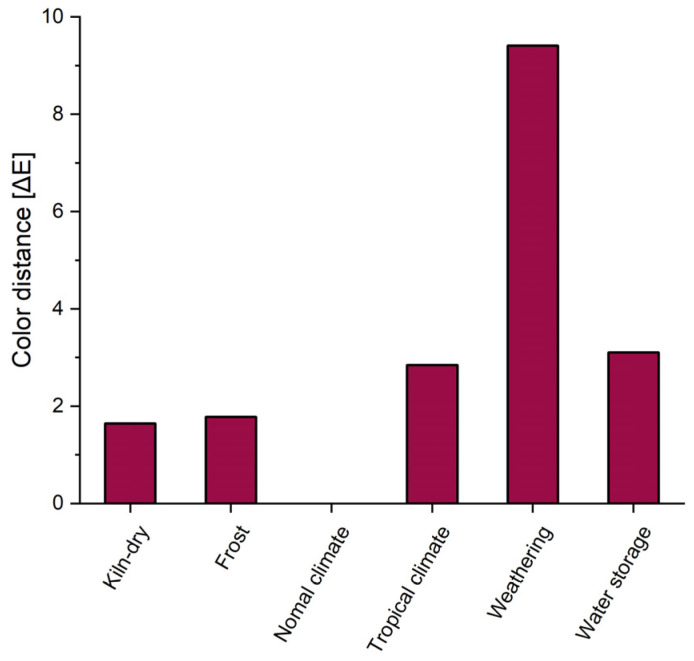
Color distance of aged WTC samples relative to the color after storage in a standard climate.

Figure 6 shows the color distance of the samples after aging, calculated using Formula (2) relative to the coloring under standard climate conditions for each case. As already determined from the individual L*a*b* values, the color of the test specimens deviates from the standard climate at all levels of aging. The weathered test specimen particularly stands out. A similar increase in ΔE was found, for example, by Fabiyi et al. [66,67] and Stark et al. [68], who investigated the influence of artificial weathering with a similar irradiation time on the color of WPC.

The magnitude of ΔE* can be classified according to the evaluation rules shown in Table 4. In the case of the oven-dried and frozen samples, there is only a slight difference in color, which in both cases is due to the denser packing of the molecular chains in the wood and the resulting increase in crystallinity. The samples stored in a tropical climate and in water show a moderate color difference, which can be attributed to the increase in water content in the composite. As expected, only the weathered samples show a large color difference, resulting from the previously described effects of photochemical degradation.

### 3.5. Results of FTIR Measurements

**Figure 7:**
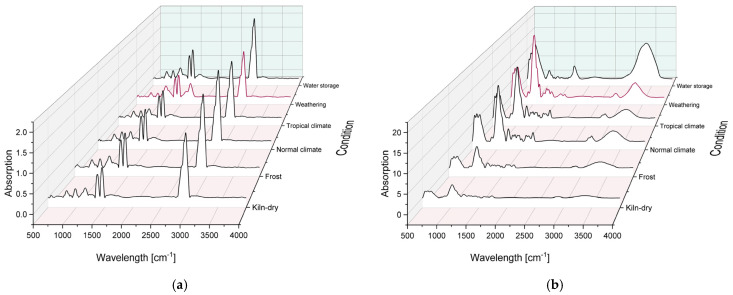
Results of FTIR measurements on (**a**) aged PP and (**b**) aged willow wood.

Figure 7 shows the measured absorption of PP (a) and willow wood (b) as a function of aging, with the measurement of the weathered sample highlighted.

In the case of PP, the region between 1800 cm^−1^ and 1700 cm^−1^ is particularly noteworthy, as it represents typical degradation products resulting from the photooxidation of polyolefins. These products include various organic compounds containing carbonyl groups (C=O), such as lactones (1785–1760 cm^−1^), esters (1750–1735 cm^−1^), ketones (1725–1715 cm^−1^), and carboxylic acids (1712–1705 cm^−1^), which collectively contribute to a characteristically broad peak in this range [32,70]. The spectra reveal increased absorption in this region compared to standard climate conditions, particularly in the weathered sample. Under the other aging scenarios, no significant changes—or only minor ones—are observed. Only the region around 3340 cm^−1^, characteristic of hydroxyl (OH) groups, shows a slight increase in the water-stored sample.

For willow wood, the most prominent feature is the change in the band at 3300 cm^−1^ (OH), which reflects variations in the wood’s moisture content. No visible difference is observed between the standard and tropical climate samples, while the band intensity increases, consistent with expectations, for the weathered and water-stored samples. In the latter case, the change is primarily due to direct water absorption, whereas in the weathered samples, hydrolysis products contribute as well, stemming from the degradation of hemicellulose and cellulose during weathering [71,72,73]. In contrast, the OH-band intensity decreases in the frost-stored and kiln-dried samples. For the kiln-dried specimens, this is due to the reduced moisture content; for the frost-stored ones, the reduction results from the crystallization of water within the material. This behavior is also reflected in the 2900 cm^−1^ band, which corresponds to C–H stretching vibrations. Although this band is generally considered stable, a reduction in intensity is observed in both the kiln-dried and frost-stored samples. This suggests structural changes in the hydrocarbon and hydrogen bonds within the cellulose, hemicellulose, and lignin components. In kiln-dried samples, this can be attributed to the breaking of hydrogen bonds, while in frost-stored samples, the presence of water in crystalline form may influence the C–H band intensity [46,72,73]. Another notable feature is the reduction in intensity at 1730 cm^−1^ in the weathered sample, indicating a loss of carbonyl groups during degradation [44,71,72,74].

The indices calculated for PP are shown in Figure 7a, each in relation to the index under standard climate conditions. The carbonyl index (C=O groups) is the most important marker for oxidative degradation during weathering and increases steadily with the duration of exposure [32,34,35]. The crystallinity index initially rises and amorphous regions degrade first, making the material more brittle. In later stages, however, this index declines again as the crystalline regions also begin to break down [32,36]. The vinylidene index (unsaturated double bonds) and the hydroxyl index (–OH groups) also exhibit characteristic aging patterns but may decrease at advanced degradation stages as these groups are further broken down or transformed [32,37,38,39]. Significant changes were only observed in the weathered test specimens, where the carbonyl index increased markedly, while the other indices showed slight declines. These changes indicate that the material is in an advanced stage of degradation following artificial weathering.

After removal from the climate chamber, the specimens appeared milky and were extremely brittle. This observation corresponds to a reduction in both crystallinity and the concentrations of vinylidene and hydroxyl groups, while the carbonyl index remained high. The degradation process negatively affects nearly all the mechanical properties of PP, including its tensile strength, impact strength, and flexibility. These changes result from the breakdown of polymer chains due to oxidative degradation. The rate of degradation depends heavily on environmental conditions: intense UV radiation, elevated temperatures, and high humidity all accelerate oxidation and, thus, material degradation. An increase in crystallinity often corresponds with increased brittleness and reduced flexibility. Therefore, a rising crystallinity index is frequently an indicator of PP aging and a decline in mechanical performance, although thermal resistance may show a temporary improvement. In particular, elongation at break serves as a useful indicator of material aging [32,75,76]. The increasingly milky appearance of the material is due to the increase in light scattering caused by growing crystalline domains, a result of the degradation of amorphous areas [36]. However, as weathering progresses, the crystallinity index eventually drops again as crystalline structures begin to deteriorate. The presence of stabilizing agents, such as UV absorbers and antioxidants, can slow down the increase in degradation indices by delaying oxidative processes [35,38,39,70,75,77].

**Figure 8:**
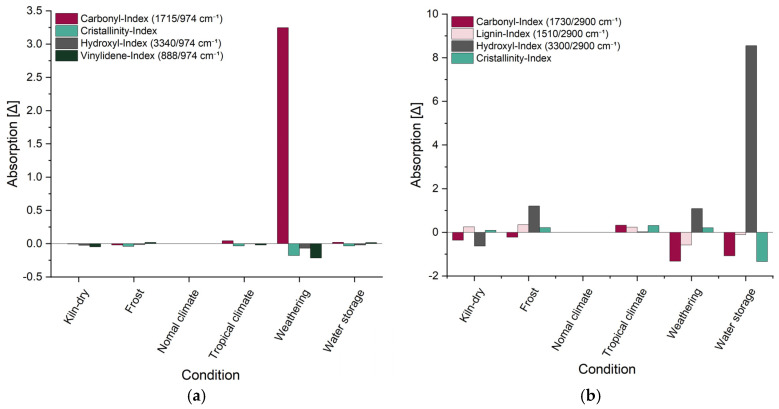
Changes in different indices of (**a**) PP and (**b**) willow wood, determined using FTIR measurements after aging; the relative change in relation to the index under standard climate conditions is shown in each case.

Figure 8b shows the indices calculated for willow wood, with the band at 2900 cm^−1^ serving as a stable reference point [43]. The carbonyl index, calculated as the ratio between the absorption bands at 1730 cm^−1^ and 2900 cm^−1^, provides insight into the formation or degradation of carbonyl groups, which may occur through processes such as hydrolysis or oxidation [40,41,42]. The lignin index, based on the band at 1510 cm^−1^, primarily reflects the photochemical degradation of lignin caused by UV radiation [42,43,44]. The crystallinity index, which uses the band at 1375 cm^−1^, indicates structural changes in cellulose, including those due to swelling or microbial activity [45,46]. The hydroxyl index, calculated using the band at 3300 cm^−1^, reflects variations in moisture content and hydrogen bonding [46]. Among the samples examined, the most significant changes in these indices were observed in the weathered and water-stored specimens. Both the carbonyl and hydroxyl indices increased, indicating ongoing oxidative processes and enhanced moisture uptake [74]. The lignin index decreased exclusively in the weathered samples, consistent with the UV-induced degradation of lignin [42,44,74]. The crystallinity index decreased during water storage due to the swelling of cellulose fibers, whereas in the weathered samples it increased, suggesting the preferential degradation of amorphous cellulose components [45,46].

FTIR spectroscopy is a useful method for detecting degradation-related chemical changes in weathered wood. The absorption band at 1730 cm^−1^ corresponds to the stretching vibrations of carbonyl groups (C=O), which are especially abundant in hemicellulose [74]. A reduction in this band’s intensity indicates the UV- and moisture-induced degradation of hemicellulose and cellulose. Concurrently, oxidation processes triggered by UV exposure and ozone may lead to the formation of new carbonyl groups, detectable near 1740 cm^−1^ in the FTIR spectrum [44,71]. The bands characteristic of lignin in the FTIR spectrum are at 1510 cm^−1^ (aromatic C=C stretching vibrations [44,74]) and 1260 cm^−1^ (C-O stretching vibrations of methoxy groups in lignin [74])). Due to lignin’s particular susceptibility to photochemical degradation, the intensities of these bands weaken or may even vanish under weathering conditions [44]. The hydrolysis of wood components and moisture uptake, which are especially relevant for hydrophilic materials like wood, can also be detected by FTIR. An increase in the intensity of the band at around 3300 cm^−1^ indicates enhanced water absorption [71]. Weathering can also lead to a reduction in the crystalline content of the cellulose. This can be detected by a reduction in band intensity at around 1375 cm^−1^ (CH bending) and 897 cm^−1^ (C-O-C stretching [44]) [74].

In summary, it can be concluded that the artificial weathering of the willow wood, analogous to the weathered PP, led to irreversible photochemical degradation within 21 days.

### 3.6. Results of the Tensile Test

Table 5 shows the absolute values of the tensile test and the corresponding coefficient of variation. Tensile strength, Young’s modulus, and elongation at break were evaluated. The low to moderate CV indicates stable results. Significant deviations from the standard climate were only found in the case of kiln-dried, weathered, and water-stored WTCs. In the case of kiln-dried samples, there was a significant decrease in tensile strength and a marginally significant decrease in elongation at break and Young’s modulus of 15% in each case. The weathered sample shows highly significant changes in relation to the standard climate, with tensile strength and Young’s modulus decreasing (−28% and −49%) while elongation at break increases (+31%). The water-stored WTCs also show a highly significant increase in elongation at break (+88%), while the Young’s modulus decreases highly significantly by 45%.

The glass transition temperature (T_g_) of PP is around −20 °C to 0 °C [78,79]; the melting range (T_k_) is between 160 °C and 170 °C [80]. For kiln-drying, the test specimens were stored at 103 °C for 24 h, i.e., at a temperature well above the T_g_. This greatly increases the mobility of the molecular chains in the amorphous areas, significantly reducing the stiffness and the ability of the material to withstand high tensile forces or elongation. Although the crystalline areas do not melt at this temperature, partial destabilization can already be assumed. This leads to a structural weakening of the molecular chains. In addition, thermal vibrations weaken the van der Waals forces between the molecular chains, which also reduces the tensile strength and the modulus of elasticity. The energy-absorption capacity of the material under load is reduced and the elongation at break is reduced. In summary, the elongation at break is reduced due to the reduced cohesion in the amorphous areas and the destabilization of the crystalline areas, as the material’s ability to deform plastically is severely impaired. The weakening of the intermolecular forces means that the material is less resistant to tensile stress and the tensile strength decreases. The increased mobility of the molecular chains leads to reduced stiffness and a decrease in the modulus of elasticity [78,79].

At temperatures around 100 °C, the first softening reactions of hemicellulose and lignin occur in wood, reducing the stiffness of the cell wall and decreasing the modulus of elasticity. Thermal mobility increases at higher temperatures, weakening the intermolecular bonds between the molecular chains of the wood components and reducing tensile strength. In addition, the thermal energy destabilizes the hydrogen bonds in the cell wall, reducing the cohesion between the molecular chains. Water bound in the cell wall evaporates, reducing both the flexibility and stability of the cell wall structure. As a result, the wood loses its ability to deform plastically, which in turn leads to stress areas and microcracks, and thus to a more brittle fracture behavior [23,81].

Storage in water, on the other hand, had no significant effect on the polypropylene, either due to temperature or humidity, especially since the temperature was identical to that of standard climate conditions. It can therefore be assumed that the increase in elongation at break and the decrease in the modulus of elasticity are mainly due to the properties of the wood in the composite. Hemicellulose in particular, but also cellulose, bind water to hydrogen bonds, which leads to an increase in the mobility of the molecular chains in the amorphous areas. This enables increased plastic deformation, which increases the elongation at break of the wood. At the same time, the intermolecular bonds between the molecular chains, which are responsible for the stiffness of the wood, are weakened by the storage of water in the cell wall matrix. The cell wall swells, and the structural cohesion of the wood components is impaired. The ability of the wood to deform elastically is reduced and the modulus of elasticity decreases [23,81].

The results of the weathered test specimens confirm the previous assumption that the changes in properties with increasing water content are mainly due to the influence of the wood. The wood moisture content of the weathered test specimens at the time of testing was lower than the moisture content after water storage. Nevertheless, there was a highly significant increase compared to the standard climate. As with water storage, this results in an increase in elongation at break and a reduction in the modulus of elasticity. Unlike water storage, weathering also causes a highly significant decrease in tensile strength. As described in Section 3.5, after weathering, the material is in a late stage of degradation, with the PP matrix being particularly affected, as well as the wood fibers in some cases. In contrast to water storage, the matrix is almost completely destroyed as a result of artificial weathering, meaning that force transmission in the sense of composite material theory is no longer possible. The photochemical degradation of the lignin also reduces the wood’s resistance to tensile stress. Similar reductions in tensile strength and the modulus of elasticity were demonstrated, for example, by Badji et al. [82] and Mohammed et al. [83], as a result of the weathering of natural fiber-reinforced polymers.

### 3.7. Results of the Impact Bending Test

Table 6 shows the absolute values and the CV of the impact bending test. All the results show a uniformly high variance of >20, which indicates a comparatively large variation in the results. Due to the high CV of approximately 25% on average, a larger number of test specimens is therefore recommended for WTCs in future tests based on the heterogeneous structure of the test specimens. This could not be achieved in the case of the investigations carried out here due to the limited amount of willow wood fabric available. Nevertheless, the results obtained can at least indicate trends regarding the effect of aging on WTC. Only the kiln-dried and weathered test specimens show a marginally significant change in impact bending strength compared to the standard climate (−24%; −26%), while the water-stored test specimens show a significant change (+75%). However, no significant changes were observed after storage in freezing and tropical climates.

The impact strength decreases in the case of kiln-dried test specimens, which is due to both thermal dehydration and the softening of lignin and hemicellulose. The cohesive properties of the wood decrease, its ability to absorb energy is reduced, and the wood becomes brittle [81]. At the same time, the softening of the amorphous portion in the PP reduces the ability to absorb energy through elastic or plastic deformation. The matrix thus becomes more brittle and stiffer and, like wood, is less able to absorb energy through structural deformation [78].

The increase in impact strength under frost conditions can be attributed to the stiffening effect of frozen water within the wood’s cell walls and the resulting enhancement of cohesion between wood components [81,84]. Although the material becomes more brittle, the stiffening improves its capacity to absorb impact energy. At temperatures near the lower end of PP’s glass transition range, the mobility of molecular chains in the amorphous regions is significantly reduced, while the molecular structure remains intact. This leads to an effect similar to a reinforced crystalline structure, increasing stiffness and, consequently, energy absorption. In contrast, weathered specimens exhibit a marked reduction in impact strength due to advanced photochemical degradation and the resulting breakdown of the composite structure [85]. Lastly, water absorbed during storage also leads to a significant increase in impact strength compared to standard climate conditions. Kohl et al. [86] observed similar behavior in impact bending tests and attributed it to increased energy absorption by the water-containing composite.

## 4. Conclusions

This study examined the influence of various climatic conditions on the chemical and mechanical properties of WTC. To this end, the base materials of the composite—PP and willow wood—as well as the composite itself were subjected to different aging scenarios, and the resulting thermo-hygroscopic effects were analyzed.

The results show that thermo-hygroscopic influences—particularly during weathering—have a significant impact on WTC properties, mainly due to the hygroscopic nature of the wood. Most of the mechanical properties assessed are negatively affected by both increases and decreases in moisture content, as well as changes in ambient temperature. However, this does not apply to all characteristics. Depending on the type of test, especially the nature and direction of the applied load, some properties even show improvements under certain aging conditions.

This raises the question of how these findings might be specifically leveraged in practical applications. A central challenge is that the effects studied rarely occur in isolation but typically act simultaneously or in succession. For instance, while impact tests show improved performance at sub-zero temperatures, real outdoor conditions—combining UV radiation and rainfall—can lead to permanent degradation of the composite. Similarly, the increased resistance to sudden loads following water storage is of limited practical value if repeated swelling and shrinkage of the wood cause delamination and cracking, resulting in lasting damage.

Although not all forms of aging had a detrimental effect on the WTCs examined, the overall conclusion is that the thermo-hygroscopic influences studied contribute to the medium- to long-term degradation of the composite material.

For use in unprotected outdoor environments—whether for esthetic or functional purposes—the findings indicate that stabilization against weather-induced degradation is essential. Structural protective measures, such as sealing exposed wooden edges to prevent moisture ingress, represent one possible approach. In addition, stabilizing the polymer matrix, for example, through the use of UV stabilizers, is also a suitable option.

## Figures and Tables

**Table 1:** Aging parameters for WTCs.

Aging Conditions	Parameters	Time	Reference
Kiln-drying	103 °C	24 h	[29]
Frost	−18 °C	24 h	-
Standard climate	23 °C/50% r. h.	168 h	[26]
Tropical climate	27 °C/65% r. h.	168 h	[26]
Weathering	50 °C + UV (5 h) 23 °C + Rain (1 h)	504 h	[28]
Water-storage	23 °C	24 h	[27]

**Table 2:** Wavelengths used to calculate different indices based on FTIR measurements.

Material	Index	Wavelength	References
PP (reference wavelength: 974 cm^−1^)	Carbonyl Index	1715 cm^−1^	[32,34,35]
	Crystallinity Index	998 cm^−1^	[32,36]
	Vinylidene Index	888 cm^−1^	[32,37]
	Hydroxyl Index	3340 cm^−1^	[32,38,39]
Willow (reference wavelength: 2900 cm^−1^)	Carbonyl Index	1730 cm^−1^	[40,41]
	Lignin Index	1510 cm^−1^	[42,43,44]
	Crystallinity Index	1375 cm^−1^	[45,46]
	Hydroxyl Index	3300 cm^−1^	[41,47]

**Table 3:** Absolute L*a*b* values of the color measurements of WTC and corresponding CV.

Aging Conditions	Red–Green		Yellow–Blue		Light–Dark	
	a*	CV (%)	b*	CV (%)	L*	CV [%]
Kiln-drying	9.19	3	25.08	3	67.20	2
Frost	8.23	4	24.00	5	68.50	1
Standard climate	8.98	9	25.60	7	68.75	1
Tropical climate	10.16	1	28.18	1	68.87	0
Weathering	7.64	50	16.36	43	69.91	9
Water storage	10.17	3	25.57	5	65.89	1

**Table 4:** Assessment rules for the size of ΔE based on [69].

Magnitude of ΔE	Classification
0.2 > ΔE	No color difference
0.2 < ΔE < 2	Slight color difference
2 < ΔE < 6	Moderate color difference
6 < ΔE < 12	Significant color difference
12 < ΔE	Different colors

**Table 5:** Absolute values of the tensile test and the corresponding CV.

Aging Conditions	Tensile Strength [MPa]	CV (%)	Elongation at Break [%]	CV (%)	Young’s Modulus [MPa]	CV (%)
Kiln-dry	38.08	11	0.87	18	4420.83	16
Frost	43.81	4	0.98	10	4893.78	13
Standard climate	44.81	5	1.02	9	5215.44	12
Tropical climate	42.76	11	0.95	9	5301.60	3
Weathering	32.05	4	1.34	14	2665.34	10
Water storage	43.73	7	1.92	9	2894.42	12

**Table 6:** Absolute values and CV of the impact bending test.

Aging Conditions	Impact Bending Strength (kJ/m²)	CV (%)
Kiln-dry	12.77	27
Frost	18.59	28
Standard climate	16.70	26
Tropical climate	17.72	27
Weathering	12.40	22
Water storage	29.25	23

## Data Availability

The data presented in this study are available on request from the corresponding author.

## Abbreviations

The following abbreviations are used in this manuscript:

WTC	Wood–Textile Composites
UV	Ultraviolet
μ-CT	Micro Computer Tomograph
FTIR	Fourier Transform Infrared Spectroscopy
PP	Polypropylene
WPC	Wood–Plastic Composites
CV	Coefficient of Variation
MC	Moisture Content
